# EFCAB2 is a novel calcium-binding protein in mouse testis and sperm

**DOI:** 10.1371/journal.pone.0214687

**Published:** 2019-04-01

**Authors:** Hossam H. Shawki, Yu Ishikawa-Yamauchi, Akihiro Kawashima, Yuki Katoh, Manabu Matsuda, Al-Sayed Al-Soudy, Fatma M. Minisy, Akihiro Kuno, Xiafukaiti Gulibaikelamu, Takatsugu Hirokawa, Satoru Takahashi, Hisashi Oishi

**Affiliations:** 1 Department of Anatomy and Embryology, Graduate School of Comprehensive Human Science, University of Tsukuba, Tsukuba, Japan; 2 Department of Animal Genetic Resources, National Gene Bank, Giza, Egypt; 3 Department of Comparative and Experimental Medicine, Nagoya City University Graduate School of Medical Sciences, Nagoya, Japan; 4 Faculty of Life and Environmental Sciences, University of Tsukuba, Tsukuba, Japan; 5 Institute for Advanced Medical Research, Keio University School of Medicine, Shinjuku-ku, Tokyo, Japan; 6 Department of Arts and Sciences, Faculty of Medicine, Kindai University, Osaka, Japan; 7 Pathology Department, Medical Research Division, National Research Centre, Giza, Egypt; 8 Molecular Profiling Research Center for Drug Discovery, National Institute of Advanced Industrial Science and Technology (AIST), Koutou-ku, Tokyo, Japan; Universite Clermont Auvergne, FRANCE

## Abstract

Calcium-binding proteins regulate ion metabolism and the necessary signaling pathways for the maturational events of sperm. Our aim is to identify the novel calcium-binding proteins in testis. The gene EFCAB2 (GenBank NM_026626.3, NP_080902.1) was not previously examined, and its properties and exact mechanisms of action are unknown. In this study, we performed phylogenetic and structure prediction analyses of EFCAB2, which displays definitive structural features. Additionally, the distribution, localization, and calcium binding ability of mouse EFCAB2 were investigated. Results revealed extensive conservation of EFCAB2 among different eukaryotic orthologs. The constructed 3D model predicted that mouse EFCAB2 contains seven α-helices and two EF-hand motifs. The first EF-hand motif is located in N-terminal, while the second is located in C-terminal. By aligning the 3D structure of Ca^2+^-binding loops from EFCAB2 with calmodulin, we predicted six residues that might be involved in Ca^2+^ binding. The distribution of the *Efcab2* mRNA, as determined by northern blotting, was detected only in the testis among mouse tissues. Native and recombinant EFCAB2 protein were detected by western blotting as one band at 20 kDa. *In situ* hybridization and immunohistochemical analyses showed its localization specifically in spermatogenic cells from primary spermatocytes to elongate spermatids within the seminiferous epithelium, but neither spermatogonia nor somatic cells were expressed. Moreover, EFCAB2 was specifically localized to the principal piece of cauda epididymal sperm flagellum. Furthermore, the analyses of purified recombinant EFCAB2 by Stains-all, ruthenium red staining, and by applying *in vitro* autoradiography assay showed that the physiological function of this protein is Ca^2+^ binding. These results suggested that EFCAB2 might be involved in the control of sperm flagellar movement. Altogether, here we describe about EFCAB2 as a novel calcium-binding protein in mouse testis and sperm.

## Introduction

Mammalian spermatogenesis, which occurs in the seminiferous tubules within testis, is the process of continuous production of sperm throughout adulthood life in most mammals. This process is divided into three phases, mitosis, meiosis, and post-meiosis, which ends up with the release of the immotile sperm into the seminiferous lumen, the so-called testicular sperm [1]. Testicular sperm are impotent to fertilize eggs until they are capacitated to acquire their capability along passing through the male and female reproductive tracts [2]. Capacitation is visualized by hyperactivation of sperm motility and their ability to undergo acrosome reaction [3]. It is well known that these maturational events are regulated by Ca^2+^-signaling pathways [4]. Any error interferes the Ca^2+^-signaling is likely promoting infertility. Ca^2+^ ions reversibly bind to Ca^2+^-binding proteins to fulfill various cellular activities [5]. However, the exact mechanism of Ca^2+^ ion signaling participating in the regulation of flagellar beating, as well as the acrosome reaction of mammalian spermatozoa, is so far unclear. To understand this signaling, identifying the calcium-binding proteins (CaBPs) that express in sperm are necessary.

CaBPs play an important role in controlling the sperm physiological responses to changes in Ca^2+^ ions. Many types of calcium-binding proteins have been identified, with different cellular and tissue distributions. CaBPs can contain or lack a structural EF-hand domain [6–8].

EF-hand Ca^2+^-binding proteins with a continuous helix-loop-helix topology were originally described in the structure of parvalbumin by Kretsinger and Nockolds [9]. A typical EF-hand motif contains 12 Ca^2+^-coordinating residues in the loop region with the pattern X, Y, Z, #, -X, and -Z that accommodate calcium with distinct geometries [10]. Those that belong to the EF-hand superfamily are such as calmodulin (CaM), troponin C, calbindin D-9k, parvalbumin, S100, sarcoplasmic Ca^2+^-binding proteins, and neuron-specific Ca^2+^-binding proteins [11,12]. CaM is the most known CaBPs in sperm and is found in all eukaryotic organisms [13,14]. CaM interacts with more than 300 variable target sequences and regulate many biological functions, including sperm movement [2,11,15,16,17].

In the current study, we performed an *in-silico* search for predicted Cilia and Flagella Associated Proteins (CFAP) compiled by the HUGO database [18]. Among these proteins, here we focused on an acidic protein named EFCAB2. We examined this protein’s structure, expression, localization, and calcium-binding ability. EFCAB2 is found to be a novel calcium-binding protein in mouse testis and sperm.

## Materials and methods

### Animals

Animal experiments were performed in a humane manner after the approval from Institutional Animal Experiment Committee of the University of Tsukuba. Male ICR mice at 8 weeks of age were purchased from the Institute for Animal Reproduction (Kasumigaura-shi, Japan). The mice were euthanized with carbon dioxide gas in the Laboratory Animal Resource Center at the University of Tsukuba, and all efforts were made to minimize suffering.

### Bioinformatic analyses

*In-silico* analysis was performed for predicted Cilia and Flagella Associated Proteins (CFAP) extracted from HUGO database [18]. The filtration based on the isoelectric points (pI) calculated by web-tool http://isoelectric.org/calculate.php [19] and the gene expression in mouse tissues by BioGPS database http://biogps.org/#goto=welcome [20].

The phylogenetic tree was constructed using the Neighbor-Joining method by Phylogeny.fr (http://www.phylogeny.fr/simple_phylogeny.cgi) [21]. The amino acid sequence of EFCAB2 from a range of eukaryotic species (Homo sapiens NP_115704.1, Pan troglodytes XP_016797453.1, Macaca mulatta XP_014983189.1, Bos taurus NP_001094717.1, Sus scrofa XP_003130614.1, Mus muscles NP_080902.1, Rattus norvegicus NP_001099447.1, Monodelphis domestica XP_007481581.1, Gallus gallus XP_015139478.1, and Danio rerio NP_001314837.1) was aligned using CLUSTALW [22] and colored according to the CLUSTALX scheme using JALVIEW. The EF-hands were identified via the PRTOSITE database. The potentially functional regions on the surface of EFCAB2 were identified based on phylogenetic relationships between sequence homologues. The 3D structure of mouse EFCAB2 (mEFCAB2) was constructed by ROBETTA modeling server [23] with the default parameter and visualized in PyMOL (Schrödinger, LLC). The alignment of the EF-hand motifs sequence with the 3D structure of CaM (PDB ID: 1CLL) was performed by MOE software.

### Northern blot analysis

**Fig 1:**
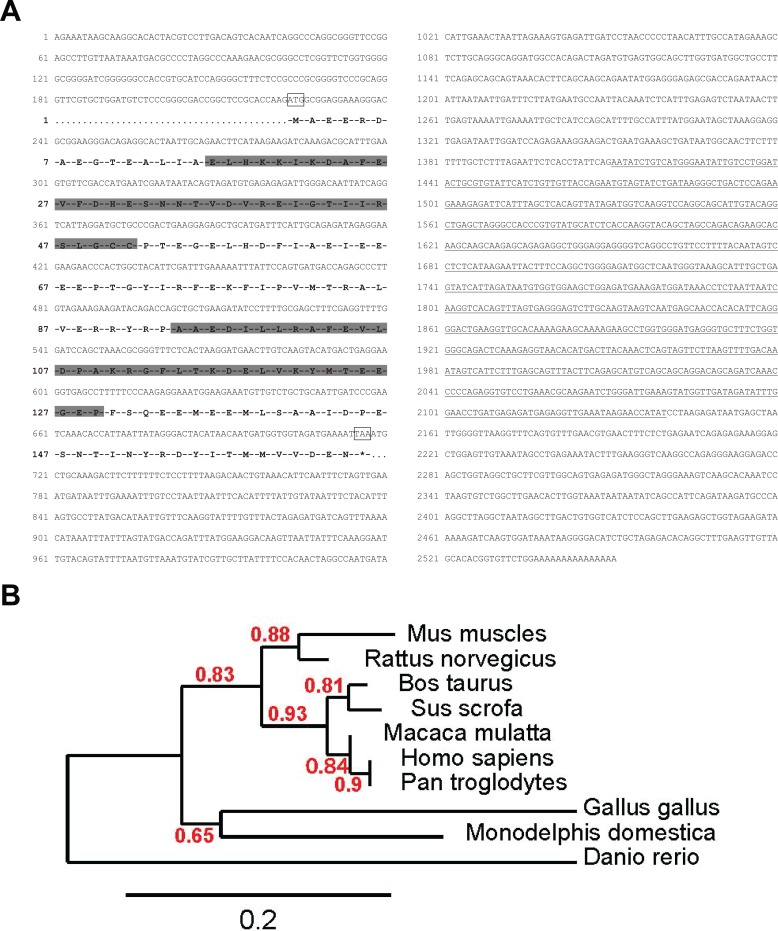
Nucleotide sequence of *Efcab2* cDNA and its conservation among species. (A) Nucleotide sequence of m*Efcab2* cDNA and deduced amino acid sequence. The entire nucleotide sequence was obtained by the NCBI database (Gene ID: 68226). Starting and stop codons are boxed. The shaded boxes indicate the sequences of the two EF-hand motif domains. The region used for DIG-cRNA probe synthesis for both Northern blot and *in situ* hybridization is underlined. The recombinant protein used for the calcium-binding analyses contained the whole entire amino acid region of EFCAB2 except N-terminal six amino acids (MAEERD). (B) Phylogenetic tree of EFCAB2 homologs. The tree was constructed by the neighbor-joining algorithm based on EFCAB2 sequences (http://www.phylogeny.fr/simple_phylogeny.cgi). Sequences were compiled from NCBI databases. A genetic distance scale and the number of times a clade occurred in the bootstrap replicates are shown.

Total RNA was isolated from mouse testes using ISOGEN (Nippon Gene, Japan), and cDNA was synthesized with a QuantiTect Reverse Transcription kit (QIAGEN; 205313). RT-PCR reaction was performed with using the primer set 5’-AAT ATC TGT CAT GGG AAT ATT GTC CTG GAT-3’ and 5’-ATA TGG TTC TTA TTT CAA CCT CTC ATC TCT-3’. Denaturation was carried out at 94°C for 30 sec with annealing at 60°C for 30 sec, and synthesis at 72°C for 2 min. The RT-PCR-amplified product was ligated into pGEM vector (Promega). After linearization by restriction enzymes, sense and antisense cRNA probes were synthesized by in vitro transcription with T7 or SP6 RNA polymerase (DIG RNA Labeling Kit; Roche Applied Science). The location of the cRNA probe in each mRNA is shown in Fig 1A.

Northern blotting was performed as per the standard protocol [24]. Briefly, total RNAs were extracted from various mouse tissues. Each 5 μg of total RNA was electrophoresed and transferred to the nylon membranes Amersham Hybond-N+ (GE Healthcare, Japan). The RNA blots were hybridized with the DIG-labeled cRNA probes according to the manufacturer's instructions (Boehringer-Mannheim Biochemica), and the visualization was performed using CDP-Star (Roche).

### Preparation of EFCAB2 recombinant protein

The recombinant protein was prepared for antigen production and for calcium-binding studies as previously described with slight modifications [25]. Briefly, *Efcab2* cDNA fragments were synthesized by RT-PCR from testis total RNAs using the primer set 5’-ACC AAG ATG GCG GAG GGA ATT CAC GCG GAA-3’ and 5’-ATT GTA TAA TGT CGA CAT TTA GTG CCT TAT-3’. The product was subsequently cloned into pGEX-6P-2 vector (GE Healthcare; Japan) through the EcoRI and SalI sites and was transformed into E.coli DH5α. Recombinant mEFCAB2-GST protein was purified using Glutathion Sepharose 4B (GE Healthcare, Japan), followed by PreScission Protease (GE Healthcare, Japan) treatment to isolate recombinant EFCAB2. The purified recombinant EFCAB2 was confirmed via amino acid sequencing.

### Production of EFCAB2 antiserum

To generate polyclonal antiserum against mouse EFCAB2, rabbits were immunized with the recombinant EFCAB2 protein. Briefly, subcutaneous injection of 1 mg of purified antigen with Freund’s complete adjuvant (Sigma) was followed by three additional booster injections of 300 *μ*g of purified antigen with Freund’s incomplete adjuvant (Sigma) at two-week intervals. Anti-mouse EFCAB2 serum was obtained from the immunized rabbits.

### Overexpression of Efcab2 in 293T cells

The coding region of *Efcab2* cDNA was amplified by RT-PCR from mouse testes using the sense primer 5’-ATA TGC GGC CGC ATG GCG GAG GAA AGG GA-3’ and the antisense primer 5’-ATA TTC TAG ATT AAT TTT CAT CTA CCA CCA TCA-3’. The product was digested with NotI/XbaI and ligated into p3xFLAG-CMV-14 expression vector (Sigma, St. Louis, MO) [26]. The 293T cells were transfected with control (empty vector) or *Efcab2*-expression plasmid using Fugene 6 transfection reagent (Promega, Madison, WI). Forty-eight hours after transfection, the protein was extracted from the cells and assigned for western blot.

### Western blot analyses

Total proteins were extracted from mouse testes or 293T cells by homogenizing in 20 mM Tris-HCl, pH 7.4, containing 1% protease inhibitor cocktail and 1% Triton X-100. The homogenates were centrifuged at 100,000 xg for 60 min at 4°C, and the supernatant was maintained at −20°C until use. The protein concentrations were determined using a Bio-Rad Protein Assay Kit II (Bio-Rad, Hercules, CA). Twenty micrograms of each extracted protein were separated by 12.5% SDS-PAGE and then transferred onto a polyvinylidene fluoride (PVDF) membrane (GE Healthcare, Tokyo, Japan) using a semi-dry transfer system (ATTO, Tokyo, Japan). The membranes were blocked in 2% bovine serum albumin (BSA) and 10% goat serum in PBS overnight at 4°C, followed by incubated with EFCAB2 antibody (1:3000 dilution in PBS) or anti β-Actin antibody (MBL PM053-7, 1:2000 dilution in PBS) for 1 h at room temperature. After three washes with PBS that contained 0.05% Tween 20 (PBS-T), the membranes were incubated with peroxidase-conjugated anti-rabbit IgG secondary antibody (Sigma, 1:2000 dilution in PBS-T), followed by three additional washes in PBS-T. Signals were developed by chemiluminescence according to the manufacturer's protocol (Millipore, Billerica, MA), and were visualized with a BAS-7000 (Fujifilm, Tokyo, Japan). The protein marker used was WIDE-VIEW prestained protein size marker III (Wako, Osaka, Japan).

### *In situ* hybridization

Fresh mouse testes were embedded in CMC compound (FINETEC), sectioned at 4 μm using a cryostat (Leica), and then incubated in PBST that contained 1 μg/ml proteinase K at 37°C for 5 min. After post-fixation with 4% PFA, the sections were rinsed in PBST three times and then hybridized overnight with 1 μg/ml DIG-labeled cRNA probe dissolved in hybridization buffer (50% formamide, 5x saline-sodium citrate, 1% SDS, 50 μg/ml heparin, and 50 μg/ml yeast RNA) at 65°C in a moist chamber. The sections were then washed in 50% formamide, 5x SSC, pH4.5, and 1% SDS at 65°C for 30 min, and in TBST three times at room temperature for 5 min each. After blocking by 0.5% blocking reagent (Roche) in TBST at room temperature for 30 min, the sections were incubated in sheep anti-DIG antibody conjugated to alkaline phosphatase in the blocking buffer at 4°C overnight. The colorization was subsequently developed by BM purple AP substrate solution (Roche Applied Science) according to the manufacturer’s instructions.

### Immunohistochemical analysis

Frozen sections of mouse testes were prepared at 5 μM thickness, permeabilized with 0.1% Triton X-100 in PBS for 20 min, and boiled for antigen retrieval in 20 mM citrate buffer (pH 6.0) for 10 min [27]. After blocking with 3% goat serum in PBST overnight at 4°C, the sections were then incubated with a 1:2000 dilution of anti-EFCAB2 antiserum as the primary antibody for 1 h, followed by incubation for an additional hour with a 1:1000 dilution of the secondary antibody Alexa Fluor 594 (Life Technologies, Gaithersburg, MD, USA) together with PNA-lectin, a FITC-conjugated peanut agglutinin (Sigma; L7381). The sections were counterstained using DAPI and observed under a fluorescence microscope (BX50/BX-FLA; Olympus, Tokyo, Japan).

### Immunocytochemical analysis

Cauda epididymal sperm were washed, diluted, and plated onto a silanized glass slide using a Shandon Cytospin 3 centrifuge. The sperm were then fixed with 4% paraformaldehyde in PBS for 5 min at room temperature. After fixation, the slides were washed with PBS, permeabilized with 0.1% Triton X-100 for 10 min, and blocked with 3% skim milk in PBST overnight at 4°C. The samples were then incubated with a 1:2000 dilution of anti-EFCAB2 antiserum as the primary antibody for an hour at room temperature. After three washes, the samples were incubated with a 1:1000 dilution of Alexa Fluor 488-conjugated secondary antibody (Life Technologies, Gaithersburg, MD, USA) for an hour at room temperature. The sections were counterstained using DAPI and visualized via fluorescence microscopy.

### Analysis of calcium-binding activity

The properties of the calcium-binding activity were investigated using the recombinant EFCAB2 protein, which covered the full length of the deduced EFCAB2 sequence except for the N-terminal six amino acids (MAEER), via Stains-all and ruthenium red. For Stains-all, first, the purified recombinant EFCAB2 protein was resolved by 12.5% SDS-PAGE, and the gels were stained with a Coomassie Brilliant Blue (CBB) kit (Nacalai Tesque, Japan); alternatively, the gels were washed overnight with 30% isopropyl alcohol, stained with Stains-all dye (Sigma) for 48 hours, and destained with isopropyl alcohol until a clear background was achieved. Second, the purified recombinant EFCAB2 protein was incubated with 5 μM Stains-all in 30% (v/v) ethylene glycol and 2 mM MOPS-KOH, pH 7.2, in the dark for 30 min, and the absorption spectra were obtained with a spectrophotometer as previously described [28]. For ruthenium red staining, the purified recombinant EFCAB2 protein was resolved by 12.5% SDS-PAGE, transferred to a PVDF membrane, and stained by Ponceau S or ruthenium red (25 mg/l of ruthenium red in 60 mM KCl, 5 mM MgCl2, 10 mM Tris-HCl, pH 7.5) for 48 hours [29].

### Radioactive calcium overlay assays

The Ca^2+^ overlay technique was performed according to Maruyama et al. [30]. In brief, proteins were resolved by SDS-PAGE and electrophoretically transferred to a PVDF membrane. After transfer, the membrane was washed three times for 20 min in freshly prepared solution (60 mM KCl, 5 mM MgCl_2_, 10 mM imidazole/HCl, pH 6.8) and then overlaid with ^45^Ca^2+^ (2 μCi/ml) for 30 min. Finally, the membrane was washed for 3 min by 50% ethanol, dried at room temperature for an hour, and exposed to KODAK XAR-5 film for 24 hours. The ^45^Ca^2+^ bound to the protein bands were detected by a Typhoon FLA 7000 computerized densitometer scanner (Fuji Film, Tokyo, Japan).

## Results

### *In-silico* exploration for calcium-binding proteins

*In-silico* analysis was performed to identify the calcium-binding proteins in testis as shown in S1 Table. We found 59 proteins predicted as Cilia and Flagella Associated Proteins (CFAP) from HUGO database. The acidic proteins have the possibility to bind calcium, therefore we filtrated these 59 proteins as acidic (pI <7), neutral (pI = 7), and basic proteins (pI >7). Results indicated that 30 proteins were found to be acidic. By looking then to their gene expression at BioGPS database, 19 out of 30 are expressed in mouse testis. Finally, among these 19 proteins, EFCAB2 and ATP2B3 were found to possess calcium-binding properties.

### Nucleotide sequence of *Efcab2* cDNA

The full nucleotide sequence of EFCAB2 cDNA was found in the NCBI database (GenBank: BC025062.1). EFCAB2 is located in chromosome 1 and contains seven exons. Only one protein coding transcript were found for EFCAB2. The cDNA clone of 2,551 bps contains a complete open-reading frame for a protein of 164 amino acids (Fig 1A). The predicted molecular weight (MW) and isoelectric point (pI) of the EFCAB2 protein were calculated to be 18.9 kDa and 4.3, respectively. The bioinformatic analyses of the conserved protein domains showed the existence of EF-hand motifs. The phylogenetic analysis of EFCAB2 by Phylogeny.fr based on sequences from ten different eukaryotic orthologs indicated extensive conservation of this protein among species ranging from Homo sapiens (NP_115704.1) to Danio rerio (NP_001314837.1) (Fig 1B). Mouse EFCAB2 shares the highest identity with human, chimpanzee, and macaque (85% each), while the lowest identity was identified with zebrafish (62%).

### Molecular structure of EFCAB2

**Fig 2:**
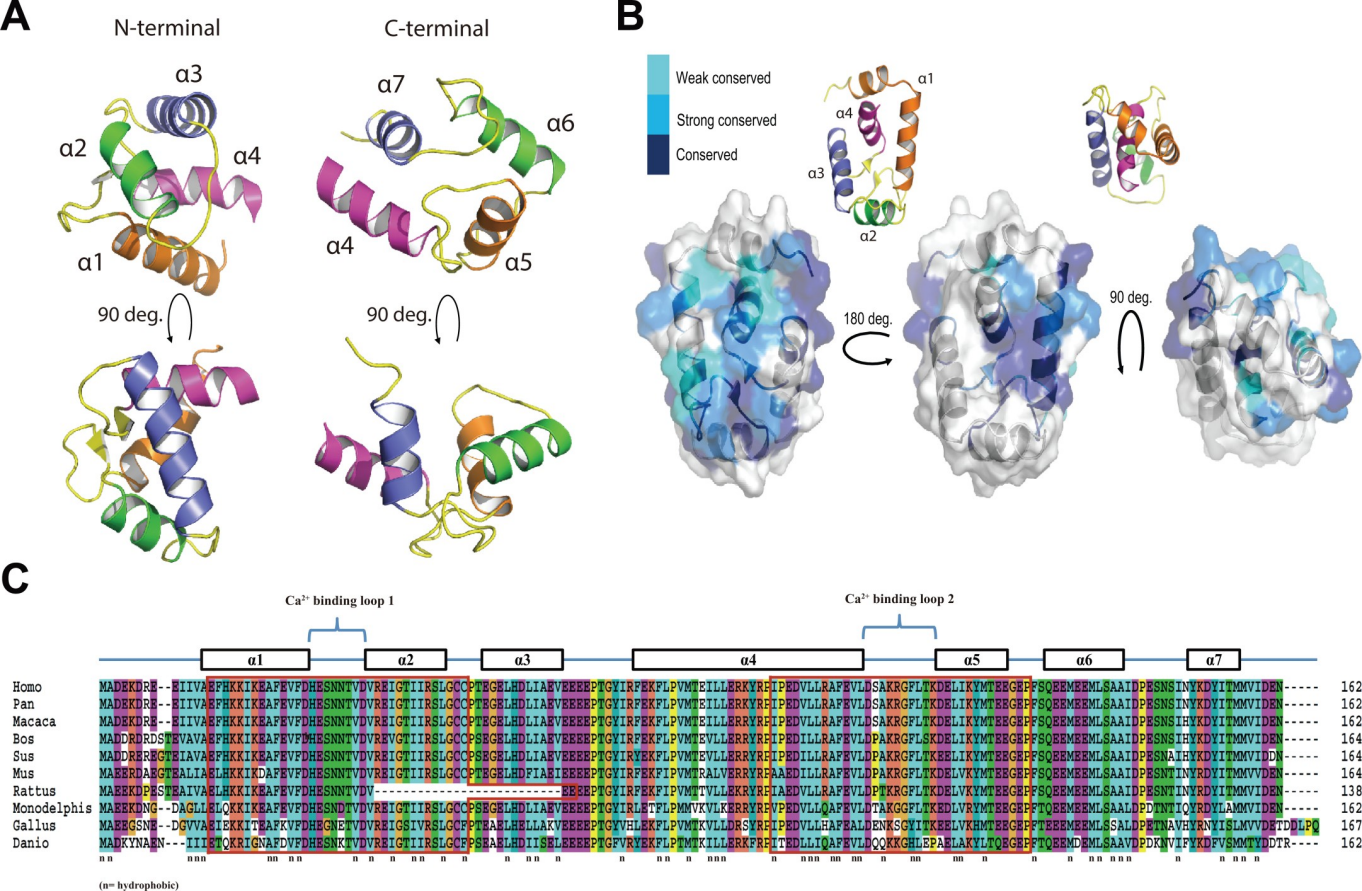
Molecular structure of EFCAB2. (A) Three-dimensional structure constructed by the ROBETTA modeling sever for mEFCAB2 protein. N-terminal (left) and C-terminal (right). (B) Surface conservation of EFCAB2 based on phylogenetic relationships between sequence homologues (blue = most conserved). (C) CLUSTALW alignment of the EFCAB2 deduced amino acids from vertebrates (colored by CLUSTALX conservation). EF-hands that were identified by PRTOSITE database are boxed. Helices α1–7 are shown based on the ROBETTA model structure of mEFCAB2. n = hydrophobic residues.

A three-dimensional (3D) model of mEFCAB2 protein was constructed by ROBETTA modeling server. The 3D structure predicted that EFCAB2 contains 7 α-helices and 2 EF-hand motifs (Fig 2A). The first EF-hand motif is located in the N-terminal (E16-C51) comprising the helix α1, Ca^2+^-binding loop, and helix α2, while the second EF-hand motif is located in the C-terminal (A94-P129) comprising a part of helix α4, Ca^2+^-binding loop, and helix α5 (Fig 2A and 2C). Sequences of EFCAB2 from eukaryotic orthologs were submitted to CONSURF [31], which predicts functionally important surfaces based on conservation. Transparent topology of the EFCAB2 surface indicated that it contains a conserved surface in a V-shaped cleft opposite the Ca^2+^-binding site formed by α1, α2, α4, and α5 (Fig 2B). Moreover, alignment of the EFCAB2 deduced amino acids from vertebrates revealed that both EF-hands possess conserved hydrophobic residues (Fig 2C).

### Identification of the calcium binding residues

**Fig 3:**
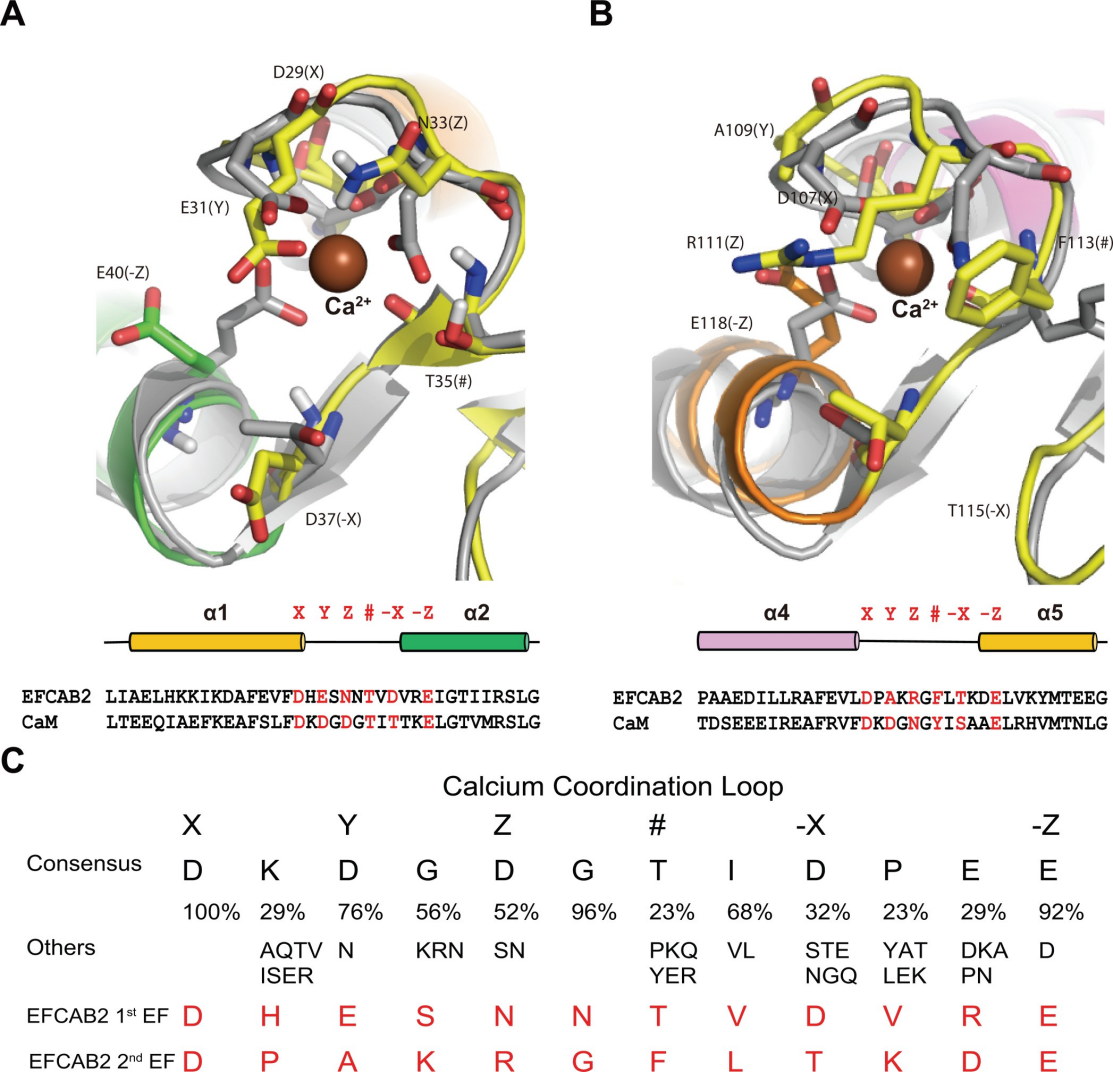
Comparison of EFCAB2 with CaM. (A) EFCAB2 Ca^2+^ binding loop for the 1^st^ EF-hand alignment with CaM. (B) EFCAB2 Ca^2+^ binding loop for the 2^nd^ EF-hand alignment with CaM. Ca^2+^-coordinating residues are labeled X, Y, Z, #, -X, and -Z, which are shown as yellow and orange CPK sticks from EFCAB2, while are gray CPK are sticks from CaM. The residue # coordinates Ca^2+^ with a backbone carbonyl. Amino acids sequence alignments from EFCAB2 and CaM are shown below each illustration. (D) Consensus sequence in EF-hand Ca^2+^-binding loops adapted from Gifford et al. [12] at X, Y, Z, #, -X, and -Z positions. Sequence similarity (%) and common residue substitutions are shown. The Ca^2+^-binding loops sequences for EF-hands of EFCAB2 are shown in red.

The 3D structures of the Ca^2+^-binding loops of mEFCAB2, generated by ROBETTA modeling server, were aligned with CaM in order to predict the Ca^2+^-binding residues. The results indicated that six residues labeled as X, Y, Z, #, -X, and -Z may be involved in Ca^2+^ binding (Fig 3A–3C). The first EF-hand motif possesses the residues D29, E31, N33, T35, D37, and E40, which correspond to the analogous X, Y, Z, #, -X, and -Z, respectively (Fig 3A). The second EF-hand motif possesses the residues D107, A109, R111, F113, T115, and E118, which correspond to the analogous X, Y, Z, #, -X, and -Z, respectively (Fig 3B). These residues share similarity with the common Ca^2+^-binding residues previously adapted (Fig 3C).

### Expression of EFCAB2 in mouse tissues

**Fig 4:**
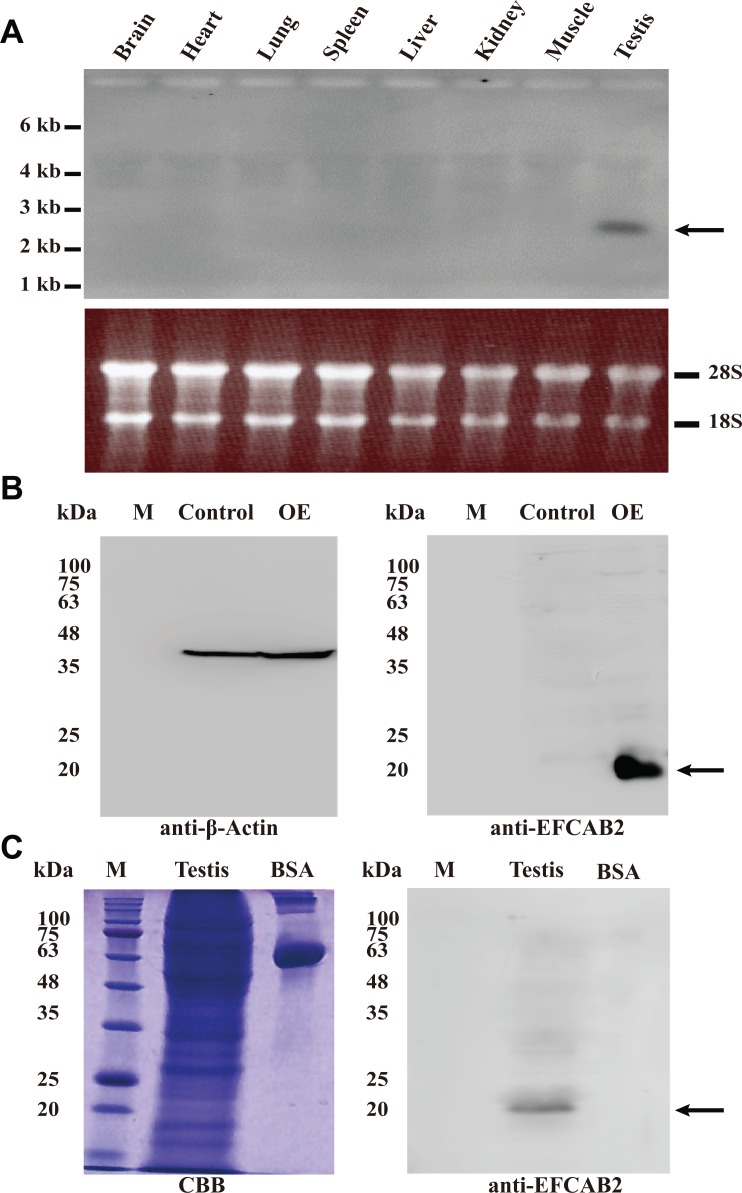
Expression of EFCAB2 in mouse testes. (A) Northern blot analysis of *Efcab2* mRNA in adult mouse tissues. Total RNAs (10 μg each) prepared from the various tissues were electrophoresed, transferred to a nylon membrane, and then hybridized with a DIG-labeled cRNA probe. The arrow indicates *Efcab2* mRNA. 28S and 18S RNA were stained as the loading controls. kb, kilobase. (B) Western blot analysis of total protein extracts from 293T cells transfected with control (P3xFLAG-CMV-14 plasmid) and EFCAB2-overexpression plasmids (OE). Immunoblotting with anti β-Actin as a loading control (left panel) or anti-EFCAB2 antiserum (right panel). The arrow indicates EFCAB2 protein band. (C) Western blot analysis of EFCAB2 protein in adult mouse testes. Total testis proteins were extracted, and 20 μg were separated by 12.5% SDS-PAGE. Left panel shows Coomassie Brilliant Blue-staining (CBB) as a loading control, while right panel shows the immunoblotted PVDF membrane with anti-EFCAB2 antiserum. The arrow indicates EFCAB2 protein band. Bovine serum albumin (BSA) was used as a negative control. M: Molecular marker (kilodalton; kDa).

The distribution of *Efcab2* mRNA was examined by northern blot analyses among various mouse tissues, including the brain, heart, lung, spleen, liver, kidney, muscle, and testis (Fig 4A). Total RNAs prepared from the various tissues were electrophoresed, transferred to a nylon membrane, and hybridized with an antisense cRNA probe coding for mEFCAB2. As shown, the *Efcab2* mRNA was detected only in the testis while not the other examined tissues. We then generated polyclonal antiserum against mEFCAB2 by collecting serum from rabbits immunized with the recombinant protein. To confirm the specificity of the produced antiserum, we overexpressed *Efcab2* in 293T cells. Total proteins were extracted from the cells 24 hours after transfection and were assigned for western blotting using the EFCAB2 polyclonal antiserum for detection (Fig 4B). The produced antibody can detect a single overexpressed band at 20 kDa, indicating its specificity. We subsequently examined the molecular weight of the native EFCAB2 protein by western blotting. Thus, total proteins were extracted from mouse testes, and the PVDF membrane was immunoblotted with anti-EFCAB2 antiserum. EFCAB2 was detected as one band at 20 kDa as shown (Fig 4C).

### Localization of EFCAB2 in mouse testis and sperm

**Fig 5:**
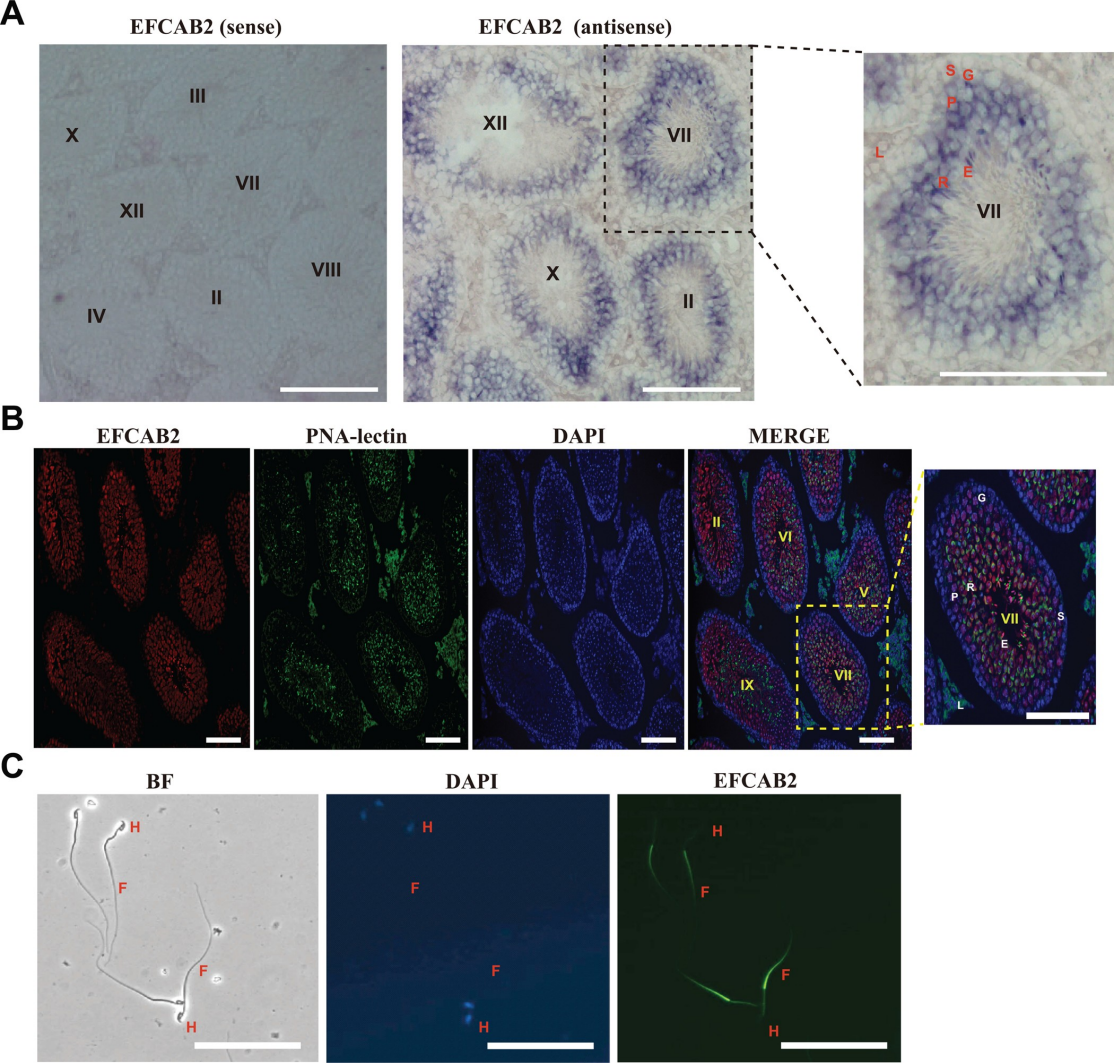
Localization of EFCAB2 in mouse testis and sperm. (A) *In situ* hybridization of adult mouse testis. Testis sections were incubated with sense (negative control) and antisense DIG-cRNA probes. (B) Immunohistochemical localization of the EFCAB2 protein in adult mouse testis. Double immunostaining with EFCAB2 (red) and the acrosomal marker PNA-lectin (green). Nuclei were counterstained with DAPI (blue). (C) Immunocytochemical localization of EFCAB2 protein in cauda epididymal sperm. Immunostaining with EFCAB2 (green) and nuclei were counterstained with DAPI (blue). L, Leydig cell; S, Sertoli cell; G, spermatogonium; P, pachytene spermatocyte; R, round spermatid; E, elongating spermatid; H, head; F, flagellum; Roman numerals, epithelial stages; BF, bright field; Scale bar, 100 μm.

The localizations of *Efcab2* mRNA and EFCAB2 protein in adult mouse testis were examined via in situ hybridization and immunostaining analyses, respectively. *Efcab2* mRNA was specifically detected in germ cells within the testis sections but not in somatic cells. The *Efcab2* mRNA expression at seminiferous stage VII of the epithelium was detected from primary spermatocytes to elongate spermatids (Fig 5A). However, the expression was not detected in spermatogonia. Antisera against EFCAB2, which was obtained from the immunized rabbits, was utilized to examine the distribution of EFCAB2 protein in the testis and sperm. Immunostaining analyses confirmed the expression pattern of EFCAB2 protein in the mouse testis. The EFCAB2 signal was observed from primary spermatocytes to elongate spermatids (Fig 5B). Furthermore, indirect immunofluorescence of mouse caudal epididymal sperm demonstrated that EFCAB2 was specifically localized to the principal piece of sperm flagellum (Fig 5C).

### Calcium-binding activity of EFCAB2

**Fig 6:**
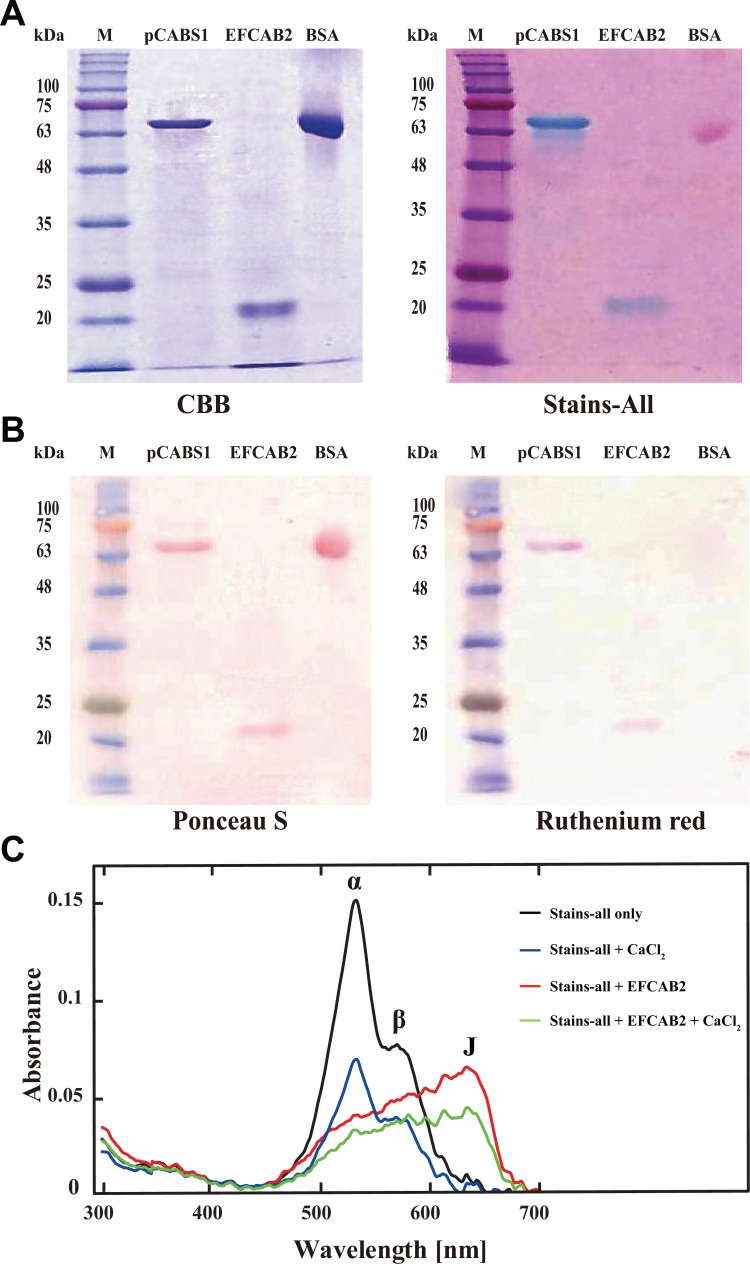
Calcium-binding activity of the recombinant EFCAB2 protein. **(**A-B) Purified recombinant EFCAB2 (5 *μ*g) was electrophoresed on 12.5% SDS-PAGE, and gels were then stained with (A) CBB and Stains-all, or the electrophoresed recombinant EFCAB2 was transferred from the gel onto PVDF membranes and stained with (B) Ponceau S and ruthenium red. pCABS1 was used as a positive control, while BSA was employed as a negative control. M: Molecular marker (kilodalton; kDa). **(C**) Absorption spectra of complexes of Stains-all with EFCAB2. Purified mouse recombinant EFCAB2 (5 μg) was incubated with 5 μM Stains-all in the absence or presence of 10 mM CaCl_2_ in the dark for 30 min, followed by measurement of absorption spectra. Black line and red line indicate Stains-all spectra in the presence of 0 and 5 μg of the recombinant EFCAB2, respectively. Blue line indicates Stains-all spectrum in the presence of 10 mM CaCl_2_. Green line indicates Stains-all spectrum in the presence of 5 μg of the recombinant EFCAB2 and 10 mM CaCl_2_. The addition of Ca^2+^ reduced the J band by displacing the dye from the recombinant protein.

Recombinant EFCAB2 protein was successfully purified and used for calcium-binding analyses (Fig 6). The calcium-binding activity of recombinant EFCAB2 protein was determined by the colorimetric assays using Stains-all and ruthenium red. pCABS1 was used as a positive control, while bovine serum albumin (BSA) was used as a negative control for the calcium-binding activity. In Stains-all, proteins that are very rich in acidic amino acids have the ability for calcium binding and are stained in blue or purple on gels, whereas other proteins are stained in red or pink [25]. pCABS1, EFCAB2, and BSA were separated on SDS-PAGE and stained with CBB or Stains-all (Fig 6A). The Stains-all stained the positive control (pCABS1) and EFCAB2 in blue, while the negative control (BSA) was stained in pink. Alternatively, ruthenium red is known to interact with calcium-binding proteins and shows red [25]. After SDS-PAGE, proteins were transferred onto a PVDF membrane and stained with Ponceau S or ruthenium red (Fig 6B). Ruthenium red stained pCABS1 and EFCAB2 in red, but not BSA. Furthermore, the calcium-binding activity of the recombinant EFCAB2 was also assessed by Stains-all spectrum. Calcium-binding proteins consistently formed complexes with Stains-all in the absence of Ca^2+^, which induce the J band at 600–650 nm. As shown, the recombinant EFCAB2 protein induced the J band at 600–650 nm of Stains-all (Fig 6C). The addition of 10 mM CaCl_2_ reduced the J band by displacing the dye from the recombinant protein. Overall, these results suggested that EFCAB2 protein has a calcium-binding activity.

### Analysis of direct EFCAB2 binding to calcium

**Fig 7:**
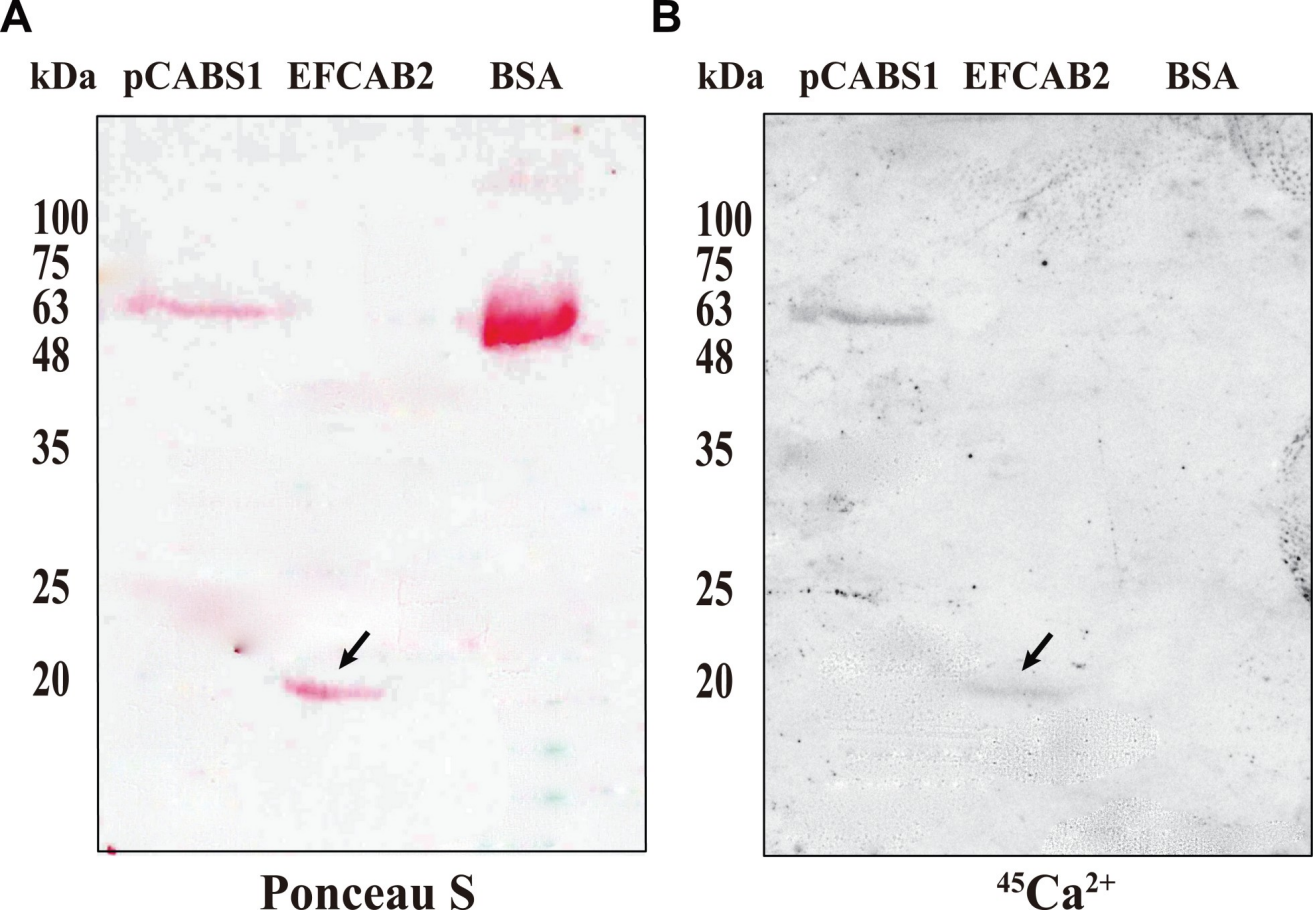
Blot overlay assay of ^45^Ca^2+^ binding to recombinant EFCAB2 protein. Protein samples (6 µg) of pCABS1 (positive control), EFCAB2, and BSA (negative control) were separated by SDS-PAGE and blotted onto a PVDF membrane. The membranes were overlaid with ^45^Ca^2+^ (2 μCi/ml) for 30 min and the ^45^Ca^2+^ binding capacity was evaluated by autoradiography. (A) Ponceau-stained PVDF membrane before ^45^Ca^2+^ incubation; (B) Autoradiograph. *Lane 1*, pCABS1 (positive control); *lane 2*, EFCAB2; *lane 3*, BSA. Arrows indicate the EFCAB2 protein band. kDa, kilodalton.

To determine whether or not calcium ions directly bound to EFCAB2, we performed ^45^Ca^2+^ blot overlay assays (Fig 7). Recombinant EFCAB2, which was purified free from GST as described in the “Materials and Methods”, was resolved by SDS-PAGE, transferred to a membrane, and incubated with ^45^Ca^2+^. pCABS1 was used as a positive control, while bovine serum albumin (BSA) was used as a negative control. Although the transferred proteins were demonstrated by ponceau staining (Fig 7A), only EFCAB2 and the Ca^2+^-binding protein, pCABS1, specifically bound ^45^Ca^2+^, whereas BSA did not bind to radiolabeled calcium (Fig 7B). These results indicated that EFCAB2 directly bound to calcium ions.

## Discussion

Calcium ions play an important role as a second messenger of signaling pathways that regulate diverse arrays of cellular functions. Accordingly, calcium ions are involved in spermatogenic cell maturational events and are required for the successful fertilization of most organisms [32]. In order to activate the calcium ion signaling, cells occupy calcium-binding proteins (CaBPs), which bind to calcium ions and regulate their intracellular concentrations [5]. Calcium ions interact with many intercellular CaBPs that are classified into two families, the EF-hand CaBPs and the non EF-hand CaBPs [6–8]. To date, not all CaBPs that contribute to spermatogenic cell maturational events have been identified; therefore, calcium signaling remains unclear. In this study, using in silico analysis, we screened acidic proteins as calcium binding candidates and found a novel calcium-binding protein in mouse testis referred to as EFCAB2.

The EFCAB2 protein has not previously been investigated. Structural prediction, distribution, and calcium-binding ability of this protein should be the first step towards crystallization or other approaches to elucidate its cellular function. EFCAB2 is located in Chr 1 and contains seven exons. The full cDNA sequence of *Efcab2* contains 2,551 bps that coding 164 amino acids. The predicted molecular weight of EFCAB2 protein was 18.9 kDa, while the molecular mass of the recombinant and native EFCAB2 protein observed on the SDS-PAGE was 20 kDa. This difference could be explained as some highly acidic proteins show anomalous behavior migrating to a higher molecular mass than expected on SDS-PAGE as previously reported [25,33,34]. As demonstrated, EFCAB2 protein sequences share extensive conservation among different eukaryotic orthologs. The highest identity of mouse EFCAB2 was with human, chimpanzee, and macaque homologies. Thus, the investigation of mouse EFCAB2 seems to be optimal for understanding its function in humans.

EFCAB2 was found to be a member of the “EF-hand” family of CaBPs. EF-hand CaBPs have a continuous helix-loop-helix topology such as CaM, troponin C, calbindin D-9k, parvalbumin, S100, sarcoplasmic CaBPs, and neuron-specific CaBPs [9,11,12]. 3D mEFCAB2 protein was constructed and predicted that EFCAB2 contains 7 α-helices and proved the existence of EF-hand motifs. EF-hands tend to always occur in pairs so that most EF-hand CaBPs have two, four or six EF-hands. This pairing minimizes the Ca^2+^ signal required to reach protein saturation [12]. Consistently, two EF-hands were found in EFCAB2 protein. The 1^st^ EF-hand motif is in the N-terminal comprising helix α1, Ca^2+^-binding loop, and α2, while the 2^nd^ EF-hand is in the C-terminal comprising a part of helix α4, Ca^2+^-binding loop, and α5. CONSURF [31], which predicts functionally important surfaces based on conservation, reveals a conserved hydrophobic residue on both EF-hands in a V-shaped cleft opposite the Ca^2+^-binding site formed by α1, α2, α4, and α5, which suggests a Ca^2+^-dependent protein interaction sites [35,36].

CaM is the most known EF-hand CaBP found in all eukaryotic sperm [11,13–17,37]. The Ca^2+^-binding loops of mEFCAB2 were aligned with CaM to predict the Ca^2+^-binding residues. The Ca^2+^ binding residues were predicted; however, some residues were different from CaM. This differences is due to the evidence that the EF-hand motif contains 12 Ca^2+^-coordinating residues in the loop region that accommodate calcium and this sequence is flexible not fixed [10,12].

The distribution of the *Efcab2* mRNA was found to be specific to the testis. Northern blot and RT-PCR analyses did not reveal EFCAB2 transcript in other tissues. We generated polyclonal antisera against mEFCAB2 and confirmed its specificity by detecting a single overexpressed EFCAB2 band on western blot. The antisera were then used to localize the EFCAB2 expression. EFCAB2 was specifically detected in spermatocytes and spermatids within the testis sections but not in somatic cells. A similar pattern was found for *Efcab2* mRNA via *in situ* hybridization. Furthermore, EFCAB2 was specifically localized to the principal piece of sperm flagellum. Such localization has also been reported in calmodulin [14]. Calmodulin was expressed in spermatocytes and spermatids in the testis but was localized to the sperm acrosome in addition to flagellum. It is well documented that a rise in intracellular Ca^2+^ is a prerequisite for flagellar beating and hyperactivation [3,4]. Calmodulin inhibition induced the decline of sperm motility and the suppression of tyrosine phosphorylation of sperm protein during capacitation [13,14,37], suggesting a similar function for EFCAB2. Other calcium-binding proteins, in addition to calmodulin, have also been suggested to play important roles in the calcium signaling required for sperm motility according to their localization. For example, CABYR localizes to the principal piece of flagellum and has been suggested to regulate sperm hyperactivation [38]. Centrin and Calaxin localize to the dynein arms and have been suggested to regulate the dynein arms of sperm [39,40].

The calcium-binding activity of EFCAB2 protein was determined by colorimetric assays using Stains-all and ruthenium red. Stains-all dye is a cationic carbocyanine dye which is known as a sensitive probe for CaBPs by staining them in blue but other proteins in red [41,42]. The interaction of Stains-all with proteins causes changes in the absorption spectrum of the dye, resulting in different colored dye-protein complexes [41]. Stains-all shows a maximal absorption at 575 nm (α-band) and 535 nm (β-band), while in the presence of CaBPs, it shows absorption at 600–650 nm (J-band). EFCAB2 was stained in blue by Stains-all, and this binding induced only the J band. Alternatively, ruthenium red is known to interact with CaBPs and shows red [26]. The positive activity of EFCAB2 by Stains-all and ruthenium red analyses, suggests its ability for the calcium binding. Therefore, whether calcium ions directly bound to EFCAB2 was then examined by ^45^Ca^2+^ blot overlay assay. Interestingly, the results indicated that EFCAB2 directly bound to calcium ions.

In summary, the physiological function of EFCAB2 protein was identified for the first time to be a calcium-binding protein. EFCAB2 seems to have different Ca^2+^-dependent functions according to the expression pattern. In the testis, EFCAB2 is expressed in spermatocytes and spermatids, indicating it might be involved in spermatogenesis, and/or spermiation. In epididymal sperm, EFCAB2 is present in a principal piece of sperm flagellum, which suggests its involvement in the regulation of flagellar movement. The exact molecular mechanisms of EFCAB2 in germ cells must be elucidated by generating knockout alleles in near future.

## Conclusions

EFCAB2 is a novel testis-specific calcium-binding protein. The 3D structure of EFCAB2 was constructed, and the calcium binding residues were predicted. EFCAB2 contains 7 α-helices and 2 EF-hand motifs. The localizations of the mRNA and protein in adult mouse testis were specifically detected in germ cells from primary spermatocytes to elongate spermatids. The localization was also found in the principal piece of sperm flagella. The molecular weight of the native EFCAB2 protein was detected as one band at 20 kDa. Recombinant EFCAB2 was found to bind directly to calcium ions.

## Supporting information

S1 Table List of Cilia and Flagella associated proteins. (XLSX)
